# Idiopathic cocoon abdomen with congenital colon malrotation: a case report and review of the literature

**DOI:** 10.1186/s12893-020-00788-7

**Published:** 2020-06-09

**Authors:** Hui Zhou, Jinling Xu, Xingwang Xie, Jiantao Han

**Affiliations:** 1 Department of General Surgery, Wuhan Third Hospital, Wuhan, 430060 People’s Republic of China; 2 Department of Endocrinology and Metabolism, The General Hospital of Central Theater Command, Wuhan, 430070 People’s Republic of China

**Keywords:** Cocoon abdomen, Colon malrotation, Intestinal obstruction, Diagnosis, Treatment

## Abstract

**Background:**

Cocoon abdomen is a relatively rare abdominal disease characterized by the total or partial encasement of the small intestinal by a dense fibro-collagenous membrane.

**Case presentation:**

We reported an unusual case of idiopathic cocoon abdomen with congenital colon malrotation. Laparotomy and sac release were performed on the patient. The patient was no recurrence 6 months after operation. A literature review was also performed.

**Conclusion:**

Preoperative diagnosis of abdominal cocoon is difficult. A careful history, physical examination and appropriate radiology may be helpful in making a definitive diagnosis. If conservative treatment can’t relieve symptoms effectively, surgery is currently considered to be important in the management of this disease.

## Background

Cocoon abdomen is a relatively rare abdominal disease in surgical emergency that was first described by Foo et al. in 1978 [1]. It is an entity characterized by encasement of small intestine by a dense fibro-collagenous membrane. However, it can appear at any age with no gender predilection [2, 3]. Due to with insufficient specific clinical symptoms or findings, preoperative diagnosis of cocoon abdomen is very difficult [4–8]. In this study, we presented an unusual case of idiopathic cocoon abdomen with congenital colon malrotation, and reviewed some current literatures on this condition.

## Case presentation

A 58-year-old male was admitted to the General Surgery Department with a 24-h history of paroxysmal colicky abdominal pain, abdominal distention, anus stop exhaust and defecation. The abdominal pain was persistent, and he had no nausea or vomiting. His symptoms gradually worsened. He had no previous medical history of abdominal pain, chronic diseases, chronic use of medication, infectious diseases like tuberculosis and abdominal surgery. He only had a history of tension-free repair for indirect inguinal hernia.

The vitals were as follows: temperature 36 °C, pulse rate 96 beats per minute (regular), blood pressure 120/80 mmHg and respiratory rate 20 per minute. Abdominal examination showed a distended abdomen with tenderness. There were no palpable masses or gut sounds. The digital rectal examination was unremarkable.

**Fig. 1:**
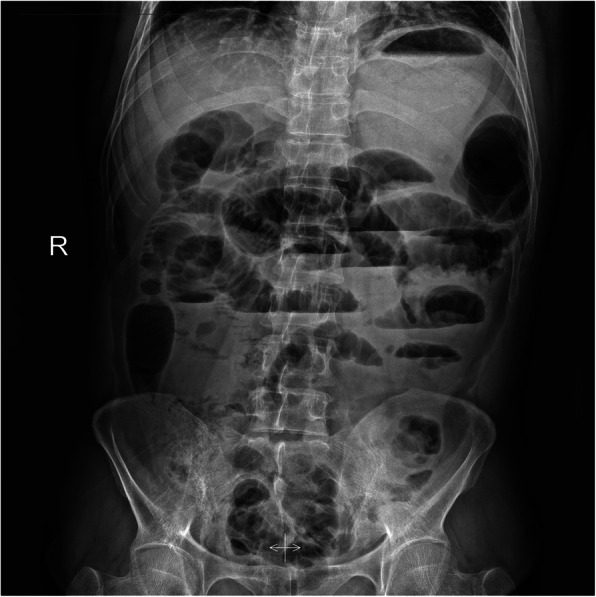
Erect abdominal x-ray at admission showing hugely dilated small bowel, with multiple air-fluid levels

**Fig. 2:**
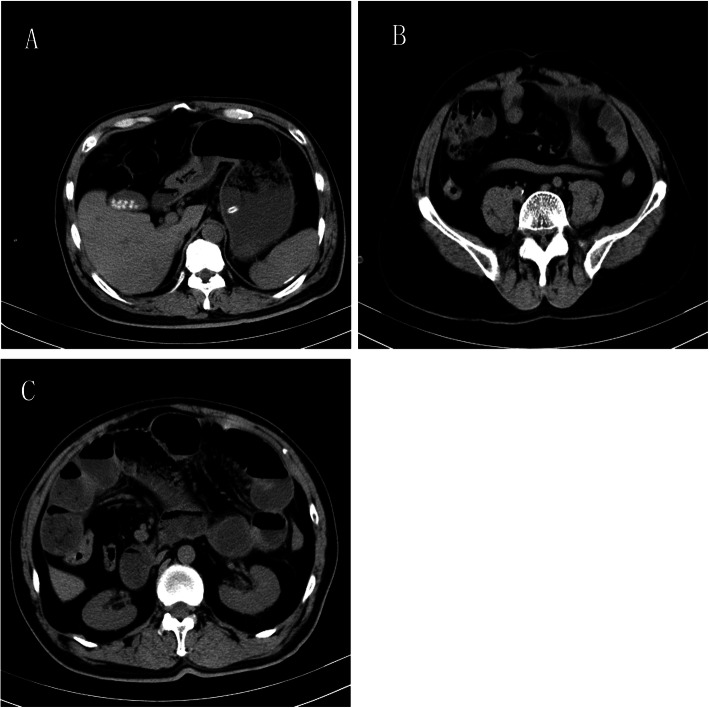
Abdominal CT at admission. **a**: Gallstones. **b**: Congenital intestinal malrotation. **c**: Small intestine obstruction with envelope wrap, fluid density around the liver can be seen, multiple gas in the intestine can be seen, accompanied by gas and liquid level

The laboratory examinations showed white blood cells 4.0 × 10^9/L, neutrophils 2.65 × 10^9/L, haemoglobin 13.2 g/dl, platelets 216 × 10^9/L, CRP 119.38 mg/L and PCT 0.32 ng/ml. Other lab findings were normal, including renal function tests, lactate and coagulation. Abdominal imaging examination was as follows: Erect abdominal X-ray detected hugely dilated small intestinal and multiple air-fluid levels in the rectum, which indicated the partial intestine obstruction (Fig. 1). Abdominal CT revealed:1. Gallstones (Fig. 2a). 2. Congenital intestinal malrotation (Fig. 2b). 3. Small intestine obstruction with envelope wrap, fluid density around the liver can be seen, multiple gas in the intestine can be seen, accompanied by gas and liquid level (Fig. 2c).

He received conservative treatments with fasting, gastrointestinal decompression, anti-infection, acid suppression and nutritional support. After 72 h, his abdominal pain and distension were aggravated. Upon physical examination, abdominal tenderness was obvious, but rebound pain wasn’t found. Abdominal CT reexamination showed dilated small intestinal was more severe than before. It indicated progressive acute intestinal obstruction. Based on clinical and radiological features, decision of laparotomy and sac release was made.

**Fig. 3:**
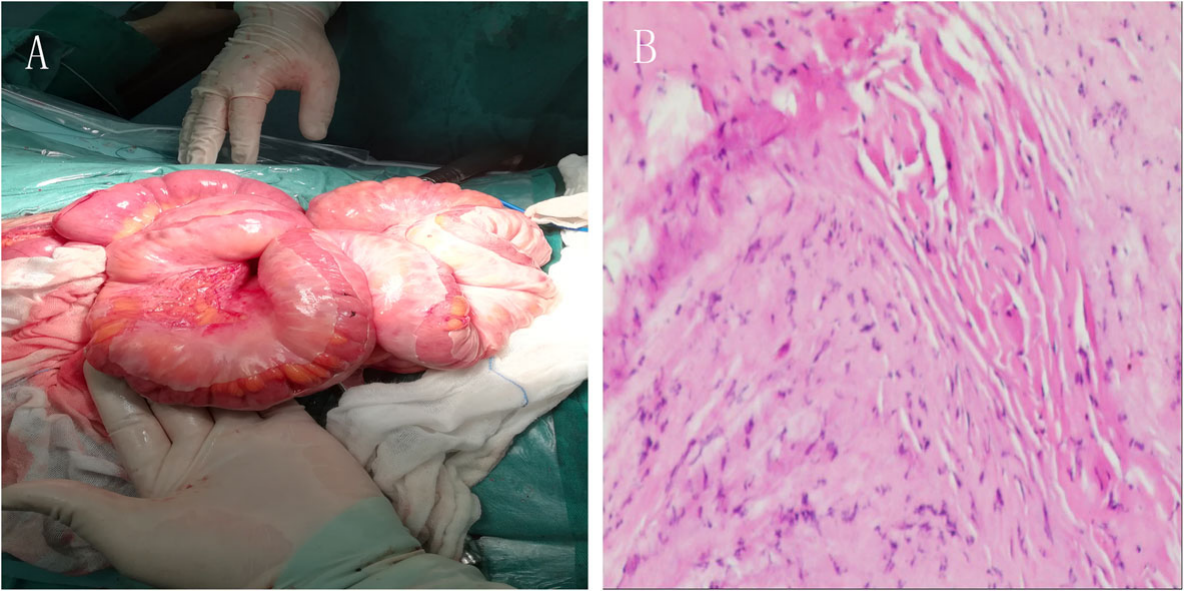
Intraoperative findings and Pathologic examination (HE × 100). **a**: A thick white membrane was found encasing the entire dilated small intestine in the abdomen. **b**: Cyst wall was composed of fibrous connective tissue with a small amount of necrosis and inflammatory cell infiltration

During exploratory laparotomy, about 200 ml light yellow ascites were found in peritoneal cavity. Then congenital colon malrotation was seen. Splenic colon and descending colon turned to the right and attached to the liver and ascending colon, and sigmoid colon transferred to right iliac fossa. At the same time, a cocoon-like fibrous membrane enclosing the small intestine (Fig. 3a). The large intestine, stomach, liver, and spleen were not affected. When the cocoon-like fibrous membrane was opened, normally dilated intestines were released. It was easy to separate the fibrous membrane from small intestine. The whole cocoon membrane was excised carefully and avoided damaging any bowel. Postoperative histopathology examination of the resected membrane showed that cyst wall was composed of fibrous connective tissue with a small amount of necrosis and inflammatory cell infiltration (Fig. 3b) .

On postoperative day (POD) 6, the patient complained of seriously abdominal pain, distension and vomiting. Bowel function was gradually recovered on POD 10 and the patient started oral intake on POD 11. He was discharged from the hospital on POD 14. A 6-month follow-up was carried out and no recurrence was detected.

## Discussion and conclusions

Cocoon abdomen is also called sclerosing encapsulating peritonitis (SEP) or chronic fibrocystic peritonitis [1]. The etiology of cocoon abdomen remains unclear. It mainly related to the following factors: 1. Congenital developmental abnormalities: absence or marked shortening of greater omentum, uterine and adnexal dysplasia, cryptorchidism, congenital small intestinal or colon malrotation and so on [9]. 2. Chronic asymptomatic peritonitis: abdominal hollow viscera perforation, endometriosis, retrograde menstruation, peritoneal dialysis, intraperitoneal drug administration (like chemotherapy), abdominal tuberculosis, cirrhosis, neoplasms with peritoneal seedings and treatment with beta blocker, etc. [10–12]. The former is classified as idiopathic, and the latter as secondary. The present case combines with congenital colon malrotation and is regarded as idiopathic cocoon abdomen.

The clinical symptoms are nonspecific and variable ranging from asymptomatic to recurrent colicky abdominal pain, nausea, vomiting, abdominal distension, abdominal ascites, constipation, anorexia, malnutrition and weight loss. Preoperative diagnosis of cocoon abdomen is really challenge and most cases have been discovered incidentally during or after the operation. Imaging examinations might be helpful for preoperative diagnosis: 1. Erect abdominal x-ray: the main manifestation is intestinal obstruction for example, hugely dilated small intestinal or multiple air-fluid levels; however, sometimes normal phenomena are also presented and it is non-specificity. 2. Transabdominal ultrasound: dilated bowel loops enclosed within a membrane, peritoneal thickening and ascites are observed. 3.Barium meal films: the characteristic serpentine structure of the distal and inner cocoon dilated small intestine can be displayed. 4. Abdominal CT: the sign on CT is shown as a serpentine configuration of dilated small intestinal loops in a U-shaped cluster. Contrast-enhanced CT is the imaging modality of choice for the diagnosis of an abdominal cocoon [13] and Preoperative Contrast- enhanced CT can diagnose it accurately [14, 15]. The main symptom of cocoon abdomen is encapsulated sign. Other associated findings may be ascites, loculated fluid, bowel wall thickening, peritoneal calcification and reactive lymphadenopathy [16]. In addition, the membrane tissues showed proliferation of fibrocytes and enrichment of collagen fiber in histopathology examination.

At present, there is no consensus on the treatment of abdominal cocoon disease. For patients with mild symptoms, conservative treatment is advocated, including fasting, gastrointestinal decompression, electrolyte disturbance correction, traditional Chinese medicine, soap-suds or sodium phosphate enema. In addition, reasonable diet and good living habits can effectively prevent the aggravation of symptoms. For patients with severe symptoms, surgical treatment is the most effective method [17, 18].

Surgical procedures included intestinal adhesiolysis and intestinal catheter separation. In idiopathic abdominal cocoon, a thin membrane surrounds the small intestinal and the capsule can be separated easily from the bowel. Excision of the small intestine is not routinely recommended unless necrosis of the small intestine occurs. On the contrary, it is usually quite difficult to separate due to a rather dense fibrous capsule with intestinal adhesion in secondary abdominal cocoon. This could be explained that why the membrane would be quite adherent in the chronic ongoing infection or inflammatory process. In the presented case the sac can be separated through an avascular plane easily which coincided with idiopathic cocoon abdomen. During surgery, the relationship between intestinal canal and mass should be carefully observed to avoid unnecessary resection. Extensive adhesiolysis for achieving complete excision of the capsule should not be carried out, so as not to damage the intestine or small intestinal vessels which lead to intestinal fistula or necrosis [19]. Furthermore, laparoscope for abdominal cocoon also achieved good results [20].

Severe complications, including intra-abdominal infection, intestinal fistula, short bowel syndrome, and bowel perforation in idiopathic cocoon abdomen are not observed. Early postsurgical activity is recommended, and the postoperative dietary guidance is also very important to their rehabilitation [21]. The diet should be gradually restored according to their gastrointestinal function. Acupuncture, physical therapy can be used to promote enterokinesia.

In conclusion, preoperative diagnosis of abdominal cocoon is difficult. A careful history, physical examination and appropriate radiology may be helpful in making a definitive diagnosis. If conservative treatment can’t relieve symptoms effectively, surgery is currently considered to be important in the management of this disease. Adhesiolysis with excision of membrane is recommended. Long-term prognosis is excellent and recurrence is rare [22].

## Data Availability

The datasets used and analyzed during the current study are available from the corresponding author on reasonable request.

## Abbreviations

CRP: C-reaction protein
PCT: Procalcitonin
CT: Computed tomography
POD: Postoperative day
